# Two-step intermediate fine mapping with likelihood ratio test statistics: applications to Problems 2 and 3 data of GAW15

**DOI:** 10.1186/1753-6561-1-s1-s146

**Published:** 2007-12-18

**Authors:** Ritwik Sinha, Yuqun Luo

**Affiliations:** 1 Division of Genetic and Molecular Epidemiology, Department of Epidemiology and Biostatistics, Case Western Reserve University School of Medicine, Wolstein Research Building, 10900 Euclid Avenue, Cleveland, Ohio 44106, USA

## Abstract

Construction of precise confidence sets of disease gene locations after initial identification of linked regions can improve the efficiency of the ensuing fine mapping effort. We took the confidence set inference, a framework proposed and implemented using the Mean test statistic (CSI-Mean) and improved the efficiency substantially by using a likelihood ratio test statistic (CSI-MLS). The CSI framework requires knowledge of some disease-model-related parameters. In the absence of prior knowledge of these parameters, a two-step procedure may be employed: 1) the parameters are estimated using a coarse map of markers; 2) CSI-Mean or CSI-MLS are applied to construct the confidence sets of the disease gene locations using a finer map of markers, assuming the estimates from Step 1 for the required parameters. In this article we show that the advantages of CSI-MLS over CSI-Mean, previously demonstrated when the required parameters are known, are preserved in this two-step procedure, using both the simulated and real data contributed to Problems 2 and 3 of Genetic Analysis Workshop 15. In addition, our result suggests that microsatellite data, when available, should be used in Step 1. Also explored in detail is the effect of the absence of parental genotypes on the performance of CSI-MLS.

## Background

With the advent of high-throughput genotyping technologies, traditional gene mapping methods, including linkage approaches, may be improved upon to realize the full potential of the wealth of genotype data available today. In particular, there has been considerable development in recent years in "intermediate fine mapping" approaches [1] in which data with dense marker maps from linkage studies are used to construct confidence sets of the disease gene locations following identification of linked regions. In addition to enhancing objectivity of the ensuing fine mapping effort by containing the disease gene locations with a pre-specified probability, a precise confidence set will reduce costs in genotyping and multiple testing.

A recent study on several competing approaches to intermediate fine mapping shows that the confidence set inference (CSI) framework is particularly promising [1]. In CSI, every genomic location within the identified broad linked region is tested against the null of being the disease gene location, and the 95% confidence set contains all the genomic locations where the null is not rejected at the 5% level. Papachristou and Lin [2] first proposed the CSI framework and implemented it by reformulating the traditional mean linkage test statistic (CSI-Mean). We recently proposed a more efficient alternative, CSI-MLS [3], by reformulating a likelihood ratio test (LRT) statistic, the maximum LOD score (MLS) [4]. The CSI procedures require knowledge of some disease-model-related parameters. When these parameters are known, CSI-MLS exhibits substantial advantages over CSI-Mean: 1) it provides more precise confidence sets for disease gene locations with correct coverage probabilities; 2) it is computationally more efficient. In the absence of knowledge of these parameters, a two-step procedure that constructs the confidence sets in the second step following the estimation of the required parameters in the first step may be adopted [5]. In this article we show that the advantages of CSI-MLS are preserved in the two-step procedures. Also investigated are the effects of three different strategies in Step 1 and the impact of the absence of parental genotype data.

## Methods

In the CSI framework using affected-sibling pair (ASP) data, each genomic location (*τ*) in a broad region with linkage evidence is tested to see whether it is the putative disease causing locus (τ*). In contrast to the traditional null hypothesis of no linkage, a new hypothesis is tested:

*H*_0*τ *_: *τ *= τ* versus *H*_*Aτ *_: *τ *≠ τ*.

A 95% confidence set for the disease gene location is constructed to contain all the genomic locations where the above null hypothesis is not rejected at the 5% level. Any traditional linkage test statistic can be reformulated to test hypothesis (1), including the mean test statistic (CSI-Mean, [2]) and the MLS statistic (CSI-MLS, [3]). Both procedures require knowledge of some disease-model-related parameters, one possibility being *z*_*i *_= *P*(τ*IBD = *i*), *i *= 0, 1, 2, where τ*IBD is the identical by descent (IBD) allele sharing between two affected siblings at the disease locus. CSI-MLS provides more precise confidence sets compared to CSI-Mean when *z*_*i *_values are known [3].

In the realistic case in which these parameters are not known *a priori*, a two-step procedure that relies on the availability of two sets of marker data on the same set of ASPs can be adopted [5]: 1) identify broad linked regions using one set of markers, termed the coarse map (e.g., microsatellite markers). Genomic locations with a nonparametric linkage statistic (KAC) [6] above 2.33 are identified as showing suggestive linkage. For the linkage peak in each region that exceeds the above cut-off point, obtain the maximum likelihood estimates of the *z*_*i *_parameters. 2) Using a set of denser markers, termed the fine map (e.g., single-nucleotide polymorphism (SNP) markers), and restricted to a region of, say, 25 cM on either side of the linkage peak, we constructed confidence sets employing CSI-MLS or CSI-Mean with the parameter estimates from Step 1. Because confidence sets so obtained may not be contiguous, we employed the smoothing scheme suggested by Papachristou and Lin [5].

Traditionally, a whole-genome linkage scan is often pursued with microsatellite markers, and then the preliminary linkage signals are followed up using a denser, usually SNP, map. It was this practice that motivated the two-step CSI procedures. However, with the advent of high-density genome-wide SNP chips, this practice may soon be replaced by a single, dense SNP map on which all individuals are genotyped. The 5 K and 10 K chips have already been successfully employed in linkage analysis. In such situations, we propose using a subset of the SNP markers as the coarse map and another mutually exclusive subset of the SNP markers as the fine map. Within each of these two maps, markers are chosen to be in linkage equilibrium with each other (*r*^2 ^< 0.02), as linkage disequilibrium between markers can lead to erroneous estimates of multipoint IBD sharing probabilities [7].

We apply our methods to Genetic Analysis Workshop 15 simulated data and the North American Rheumatoid Arthritis Consortium (NARAC) data, with the phenotype being the binary trait of the affection status of rheumatoid arthritis (RA). The microsatellite and SNP data (excluding the dense SNPs for the simulated data) on chromosome 6, containing the HLA-DRB1 locus, are used. Three strategies/settings, two using the microsatellite markers and one using the SNP markers, were compared in terms of their effects on the estimation of the disease-model-related parameters and the construction of the confidence sets. The two-step CSI-MLS and CSI-Mean were compared in terms of the precision of the confidence sets that include the DRB1 locus with a pre-determined probability. In what follows, we consider all of the 100 replicates, with a sample size of *n *(250, 500, or 750) per replicate taken to be the first *n *families of each replicate. Estimates of means, standard deviations, etc., of the parameters of interest are based on the 100 replicates of the respective sample size. "Answers" were known.

## Results

Central to the CSI framework is the knowledge of the IBD probabilities at the trait locus, *z*_0_, *z*_1_, and *z*_2_. While these are not available from the "Answers" to the simulated data, a good guess is the MLE at the HLA-DRB1 locus obtained from the chromosome 6 SNP data on all families from all available replicates. The MLE (*z*_0 _= 0.101, *z*_1 _= 0.441, *z*_2 _= 0.458) will be considered to be the true values of the corresponding parameters. The information content [8] at the trait locus was 0.97, suggesting that these estimates are very close to the true values. In what follows, we investigate the effect of the coarse map on the Step 1 parameter estimates, followed by a comparison of the performance of CSI-MLS and CSI-Mean in the two-step setting. We also study the influence of the availability of parental genotype information. Finally, both CSI-MLS and CSI-Mean are applied to the NARAC data.

### Estimates of (*z*_0_, *z*_1_, *z*_2_) at the disease gene locus

**Figure 1:**
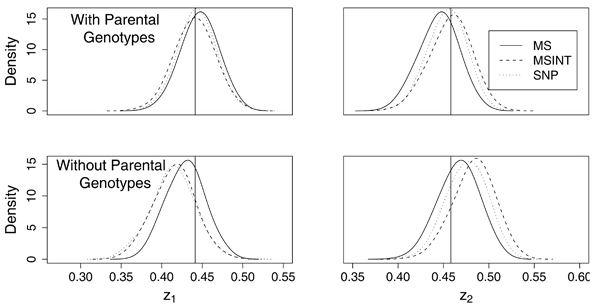
**Density of the estimates of *z*_1 _and *z*_2_**. Density of the estimates of *z*_1 _and *z*_2_, with and without parental genotypes, using 750 ASPs. The vertical lines represent the true values of the parameters.

Three estimation strategies/settings have been explored in Step 1 of CSI: 1) the MLE is evaluated at the microsatellite marker with the largest KAC score; 2) the MLE is evaluated at the location, possibly between two microsatellite markers, with the highest KAC score; 3) the MLE is evaluated with a set of SNPs that is mutually exclusive with the SNPs used in the second step as the coarse map. The three strategies are referred to as MS1, MSINT, and SNP1, respectively. The microsatellite and the SNP coarse maps contain 41 microsatellites and 292 SNPs, respectively. With parental data, the information content at the disease gene locus is 0.85 for the microsatellite map and 0.88 for the SNP map; without parental data, it is 0.56 for both. The precision of the parameter estimates were evaluated in terms of root mean squared errors (RMSEs). Table 1 provides the RMSEs of the estimates of the disease gene locations (peak of KAC scores) and of the *z*_*i *_values, with and without parental genotypes. All estimates improve with increasing sample size. With parental genotypes, SNP1 yields slightly better estimates of *z*_*i *_values than MS1 and MSINT. This finding agrees well with the information content of the maps. However, when parental genotypes are not available, MS1 yields much more precise estimates than SNP1 and MSINT. This is also reflected in the density plot of the estimates given in Figure 1. There seems to be a systematic bias in the estimates under all three setting, *z*_1 _being underestimated (for 750 ASPs, the bias was -0.01, -0.03 and -0.03 for MS1, MSINT, and SNP1, respectively) and *z*_2 _being overestimated (for 750 ASPs, the bias was 0.01, 0.03 and 0.02 for MS1, MSINT, and SNP1, respectively).

**Table 1:** Root mean squared errors of the estimates of the disease location, *z*_0_, *z*_1_, and *z*_2_

No. ASPs	Strategy	With parental genotypes				Without parental genotypes			
			
		Location (cM)	z_0_	z_1_	z_2_	Location (cM)	z_0_	z_1_	z_2_
	MS1	4.9	0.020	0.033	0.035	5.6	0.023	0.035	0.034
250	MSINT	3.8	0.020	0.033	0.033	4.8	0.024	0.041	0.040
	SNP1	2.4	0.018	0.031	0.033	4.7	0.028	0.041	0.036
									
	MS1	3.7	0.016	0.026	0.027	4.0	0.017	0.027	0.025
500	MSINT	1.9	0.016	0.026	0.024	2.3	0.017	0.037	0.035
	SNP1	1.3	0.014	0.025	0.023	3.4	0.020	0.038	0.028
									
	MS1	3.6	0.015	0.021	0.023	3.6	0.014	0.024	0.021
750	MSINT	1.5	0.013	0.022	0.020	1.8	0.015	0.034	0.033
	SNP1	1.3	0.012	0.020	0.019	2.9	0.019	0.036	0.026

### Comparison of CSI-MLS and CSI-Mean in the two-step procedure

Using the estimates of *z*_*i *_values from Step 1, CSI-Mean and CSI-MLS confidence sets are constructed with a fine map of 350 SNPs on the chromosome. Besides the three settings MS1, MSINT, and SNP1, we added the group TRUE, where the CSI confidence sets were constructed assuming the true parameter values. Mean lengths of the confidence sets, together with the empirical coverage levels, are given in Table 2. The coverage levels under TRUE are close to the nominal 95%, while they are higher than the nominal levels in all other situations. We compared the precision of the confidence sets in terms of their lengths, since the coverage is at least up to the nominal level. Under each setting, the confidence sets get tighter with increased sample size. CSI-MLS provides substantially shorter confidence sets than CSI-Mean, the effect being more pronounced when parental genotypes are not available, when the sample size is large, and when true parameter estimates are not used. For example, the reduction of the mean length is 49% (8.7 cM) with MSINT using 750 ASPs, when parental genotypes are missing. When parental genotypes are available, knowing the true values of the IBD sharing probabilities at the trait locus results in the most precise confidence sets. MSINT provides the most precise confidence sets among the three two-step procedures, with the precision close to optimal. SNP1 comes next, being only slightly less precise than MSINT. However, when parental genotypes are not available, MSINT provides much more precise intervals than all other three scenarios, with TRUE yielding the least precise intervals.

**Table 2:** Properties of 95% confidence sets constructed with CSI-MLS and CSI-Mean

No. ASPs	Strategy	With parental genotypes^a^			Without parental genotypes^a^		
			
		CSI-MLS	CSI-Mean	RR (%)	CSI-MLS	CSI-Mean	RR (%)
250	MS1	28.1 (1.00)	36.1 (1.00)	22	29.6 (1.00)	38.7 (1.00)	24
	MSINT	22.8 (1.00)	30.4 (1.00)	25	23.2 (0.98)	33.4 (1.00)	31
	SNP1	24.6 (1.00)	31.6 (1.00)	22	26.8 (1.00)	37.3 (1.00)	28
	TRUE	21.5 (0.91)	27.2 (0.95)	21	29.8 (0.96)	35.5 (0.94)	16
							
500	MS1	21.8 (1.00)	28.8 (1.00)	24	21.8 (1.00)	31.6 (1.00)	31
	MSINT	15.4 (1.00)	21.5 (1.00)	28	13.5 (0.97)	23.4 (1.00)	42
	SNP1	17.0 (1.00)	23.1 (1.00)	26	18.6 (0.99)	29.9 (1.00)	34
	TRUE	15.2 (0.92)	20.1 (0.97)	24	24.5 (0.95)	31.8 (0.98)	23
							
750	MS1	18.4 (1.00)	24.1 (1.00)	24	17.6 (1.00)	27.1 (1.00)	35
	MSINT	12.2 (1.00)	16.7 (1.00)	27	9.1 (0.95)	17.8 (1.00)	49
	SNP1	14.2 (1.00)	18.6 (1.00)	24	14.4 (0.98)	24.9 (1.00)	42
	TRUE	11.9 (0.94)	16.3 (0.96)	27	21.3 (0.97)	28.5 (0.94)	25

^a^Empirical proportions of confidence sets that contain the disease gene locus are given in parentheses. The relative reduction in length obtained by using CSI-MLS in place of CSI-Mean is given by $\text{RR}=\frac{\text{Mean length of CSI-Mean }-\text{Mean length of CSI-MLS}}{\text{Mean length of CSI-Mean }}\times 100\%$

### Application to real data

**Figure 2:**
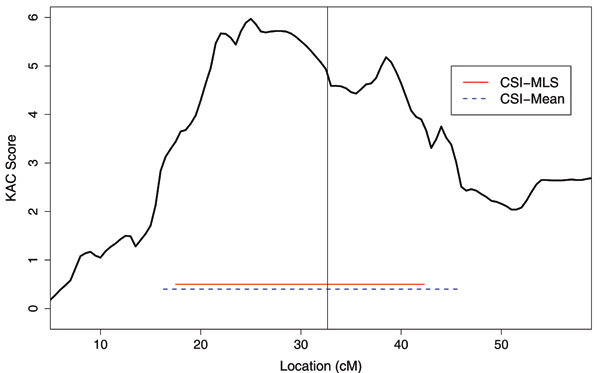
**Confidence sets provided by CSI-Mean and CSI-MLS for the NARAC data**. The KAC curve is plotted. The two horizontal lines provide the confidence intervals constructed using CSI-MLS (solid) and CSI-Mean (dashed). The disease locus is indicated with the vertical line.

The MSINT strategy appears to be the best in terms of providing precise confidence intervals for the underlying disease locus. Hence, we used this strategy with the CSI-MLS and the CSI-Mean to measure their ability in localizing the HLA-DRB1 locus on chromosome 6, a known causal locus for RA. Our sample comprised 308 of the smallest families (<9 pedigree members each). This was done to have a sample size more representative of most linkage studies, and to have nuclear families with ASPs, the setting for which the CSI-Mean and CSI-MLS have been proposed. There were 363 ASPs in the chosen families (not all independent). Figure 2 shows the KAC score for a part of chromosome 6 along with the CSI-Mean and CSI-MLS intervals and the disease locus (vertical line). CSI-MLS provides a confidence set that is 4.6 cM shorter than that provided by CSI-Mean.

## Discussion

We demonstrate here that CSI-MLS performs substantially better than CSI-Mean, with the advantage more apparent when parental genotype data are missing. This is not surprising because the trait model represents a favorable situation for CSI-MLS [3]. In a disease with no dominance genetic variance the value of *z*_1 _is 0.5. The value of *z*_1 _for RA at the HLA-DRB1 locus is 0.441, significantly different from 0.5. This difference suggests a reasonable contribution of dominance genetic variance to the total variance. CSI-MLS performs better than CSI-Mean in most situations, with the gain most substantial when dominance variance constitutes a substantial portion of the total variance of the disease [3]. The better use of allelic IBD information by the MLS statistic might explain the more pronounced advantage of CSI-MLS in the absence of parental genotype data.

When parental genotypes are absent, estimates of the disease locus IBD sharing probabilities using the MS1 strategy are superior to those using MSINT or SNP1. However, when the precision of the confidence sets are considered, MSINT provided the shortest sets while maintaining the nominal coverage. This apparent contradiction could be explained as follows. When parental information is not available, there is a bias in the estimates of *z*_*i *_values under both MSINT and SNP1 settings. However, parental data is also absent on the fine map, which leads to a similar bias in Step 2. As long as these two biases are consistent (cancel each other out), which is reasonable, the performance of MSINT and SNP1 in terms of precision of the localization can be superior to MS1. Another striking observation is that CSI-MLS confidence sets are shorter when parental genotypes are missing rather than available. For example, using MSINT with 750 ASPs, the mean length of CSI-MLS intervals is 9.1 cM with parental data compared to 12.2 cM without parental data. This is due to the former having a lower empirical coverage (95%) than the latter (100%). When microsatellite and SNP genotypes are available on the same set of families, we suggest using MSINT to estimate the parameters in Step 1 and then constructing the confidence sets using CSI-MLS on a dense SNP map in Step 2. When microsatellite data are not available, use two independent dense SNP maps in the two steps. Even though the use of SNPs in Step 1 leads to some loss in efficiency, this loss is limited. This loss can presumably be reduced by using a denser SNP map in Step 1, though one has to be careful about the LD between markers affecting parameter estimates.

CSI-MLS provides much more precise confidence intervals than CSI-Mean, yielding a reduction in length of 4.4 to 11.3 cM (16 to 49%) for the simulated data and 4.6 cM for the real data. A chromosomal region of such sizes may house hundreds of genes, and their exclusion reduces the number of candidate genes that need to be followed up by fine mapping. The problem of multiple testing is thus alleviated, and hence the power of detection of the causal locus is increased. Furthermore, budgetary constraints often limit genotyping to a fixed number of SNPs in the fine mapping stage, a smaller chromosomal region leads to better coverage of the region by the SNPs, and the causal mutations can thus be better interrogated.

## Competing interests

The author(s) declare that they have no competing interests.
